# In Vitro Production of Neutrophils Extracellular Traps Is Affected by the Lactational Stage of Dairy Cows

**DOI:** 10.3390/ani12050564

**Published:** 2022-02-23

**Authors:** Lei Xie, Osvaldo Bogado Pascottini, Jianbo Zhi, Hongzhen Yang, Geert Opsomer, Qiang Dong

**Affiliations:** 1 College of Veterinary Medicine, Northwest A&F University, Xianyang 712100, China; bill.x@nwafu.edu.cn (L.X.); zhinwafu@163.com (J.Z.); 15646702660@163.com (H.Y.); 2 Department of Reproduction, Obstetrics and Herd Health, Faculty of Veterinary Medicine, Ghent University, 9820 Merelbeke, Belgium; osvaldo.bogado@ugent.be (O.B.P.); geert.opsomer@ugent.be (G.O.); 3 Veterinary Physiology and Biochemistry, Department of Veterinary Sciences, University of Antwerp, 2610 Wilrijk, Belgium

**Keywords:** transition period, inflammation, metabolism, innate immune function, reactive oxygen species

## Abstract

**Simple Summary:**

Polymorphonuclear leukocytes (PMN) are the first line and most abundant immune cells against infection. They kill bacteria via phagocytosis, degranulation, and the formation of neutrophil extracellular traps (NETs). The formation of NETs is always accompanied by the production of reactive oxygen species (ROS). We aimed to research the NETs and ROS formation capacity of PMN in different lactational stages of Holstein cows. We also validated a model to mimic infection and inflammation to study the NETs and ROS formation capacity in vitro. Results show that the basal NETs and ROS values of PMN harvested from peripartum and lactating cows were higher than those in nulliparous heifers. The in vitro stimulation, using inflammatory products, of PMN derived from nulliparous heifers increased the production of ROS and NETs. PMN isolated from peripartum and lactating cows are primed to produce NETs and ROS, and this potentially contributes to the cows’ unfavorable inflammatory and immune status at these critical periods in their lactation cycle. The basal NETs and ROS production is lower in nulliparous heifers. Thus, they are an excellent model to mimic and study fundamental aspects of the production of NETs and ROS in vitro.

**Abstract:**

We aimed to research the neutrophil extracellular traps (NETs) and reactive oxygen species (ROS) formation capacity of polymorphonuclear cells (PMN) during different lactational stages of Holstein cows. We also aimed to validate a model which could mimic infection and inflammation in vitro by adding increasing concentrations of lipopolysaccharide (LPS) and phorbol 12-myristate 13-acetate (PMA) to PMN suspensions isolated from nulliparous heifers and evaluate their capacity to produce NETs and ROS. In 3 replicates, we collected blood from nulliparous heifers (*n* = 3), cows at the end of gestation (*n* = 3), early postpartum (*n* = 3) and in mid-lactation (*n* = 3) in which PMN were isolated. The production of ROS in PMN were assessed using the 2’,7’-Dichlorofluorescein diacetate method, while the SYTOX Orange and Quant-iT™ PicoGreen dsDNA ultra-sensitive nucleic fluorescent acid staining methods were applied in order to quantitatively analyze the formation of NETs. Statistical analyses were performed via linear regression models using the replicate as a random. ROS values of PMN harvested from peripartum cows were 1.3 times increased compared with those in nulliparous heifers (*p* < 0.01). Compared with nulliparous heifers, the production of NETs by PMN isolated from mid-lactation and postpartum cows was 2.1 and 2.5 times higher (*p* < 0.01), respectively. In 3 replicates, in vitro stimulation of PMN isolated from nulliparous heifers (*n* = 3) with LPS linearly increased the production of ROS and NETs (R^2^ = 0.96 and 0.86, respectively). Similarly, when PMN isolated from nulliparous heifers were stimulated with PMA, a linear increase in the production of ROS (R^2^ = 0.99) and NETs (R^2^ = 0.78) was observed. The basal NETs and ROS production is lower in nulliparous heifers. Thus, they are an excellent model to mimic inflammation and study fundamental aspects of the production of NETs and ROS in vitro.

## 1. Introduction

In their transition period, dairy cows are confronted with some very typical challenges that affect their basic physiology. The most important are negative energy balance (NEB), systemic inflammation, insulin resistance, and subclinical hypocalcemia [1]. Some of these features are already present before calving, but they are exacerbated in the immediate postpartum period. Despite not being fully evidenced yet, it has been suggested that there are multiple direct and indirect associations among these typical adaptive features leading to immune dysfunction in the transition period [2,3], resulting in an overall high disease incidence in the postpartum period [4]. The underlying mechanism may be that NEB metabolites (e.g., non-esterified fatty acids (NEFA) and β-hydroxybutyrate (BHB)) trigger oxidative stress concomitantly with a systemic pro-inflammatory state, and this multifactorial condition has repercussions on the innate immune function [5]. Interestingly, metabolic inflammation and pathogen-induced inflammation are not the same. In transition dairy cows, for example, the inflammatory stimulus triggered by fat mobilization on immune cells occurs earlier (and most likely differently) than the influence of pathogens on tissues, such as bacterial infection in the postpartum uterus [6]. Therefore, research to further elucidate the full pathogenesis of maladaptation to the transition period and how to monitor the impact of metabolites and endotoxins such as lipopolysaccharide (LPS) on immune cells is of great importance for the current dairy industry.

Polymorphonuclear neutrophils (PMN) are the primary effector cells of the innate immune system and are the major contributors to the defense that is substantiated during inflammatory processes [7]. Multiple studies demonstrated the association between NEB, systemic inflammation, and PMN phagocytosis and degranulation [8,9]. In this context, the formation of neutrophil extracellular traps (NETs) has been considered an important defense mechanism of the innate immune system, consisting of the release of net-like fiber structures outside of the cell to capture pathogenic microorganisms [10,11]. However, in addition to their powerful anti-pathogen effect, NETs may also directly or indirectly damage host tissues. Thus, the formation NETs by circulating PMN plays an essential role in the host’s early immune response and has been confirmed to contribute to inflammatory reactions in dairy cows [12,13]. Interestingly, the formation of NETs is always accompanied by reactive oxygen species (ROS) production, and therefore, ROS may be an indirect way to evaluate the production of NETs in vitro. Moreover, the production NETs and ROS can be considered potential indicators or biomarkers to monitor (metabolic) inflammation and immunosuppression [14]. Though, it is not fully elucidated yet whether the capacity to produce NETs and ROS by circulating PMN is different in dairy cows during the transition phase in comparison to other periods in the lactation cycle or even in nulliparous heifers.

We hypothesized that the NETs formation capacity of circulating PMN is variable according to the cow’s lactation cycle. Therefore, the goal of the present study was to quantify the formation of NETs and ROS by PMN isolated from dairy cows in different physiological states and to perform a validation study to standardize the accuracy and repeatability of the assessment of the formation of NETs and ROS in vitro.

## 2. Materials and Methods

### 2.1. Reagents and Materials

Trypan blue, Triton X-100, 2,7-dichlorofluorescein diacetate (DCFH-DA), lipopolysaccharide from E. coli O55:B5 (LPS) (L2880), and phorbol 12-myristate 13-acetate (PMA) (P8139) were purchased from Sigma-Aldrich (Shanghai, China). SYTOX orange dye (S11368), transparent 24-well plates, and Quant-iT™ PicoGreen dsDNA Assay kit (P7589) were purchased from ThermoFisher Scientific Technology Co., Ltd. (Shanghai, China). Roswell Park Memorial Institute (RPMI) 1640 dry powder and phenol red-free RPMI1640 liquid medium were purchased from Gibco Life Technologies Co., Ltd. (Shanghai, China). Polylysine and Giemsa stain kits were purchased from Jiangsu Beyotime Biotechnology Co., Ltd. (Nanjing, China). Black 96-well plates were purchased from Shanghai Jingan Biological Technology Co., Ltd. (Shanghai, China).

### 2.2. Ethical Approval and Sample Size Calculation

All of the animal experimental procedures were approved by the Animal Care Commission of the College of Veterinary Medicine, Northwest A&F University, China (NWLA-2021-062). Cows for the present experiment were provided by the Aohua Modern Animal Husbandry (E 107°55′31.9476″, N 34°11′30.8868″), Meixian, China.

Regarding the number of animals used in the present experiment and since no previous study compared the production of NETs in dairy cows at different lactational stages, our sample size calculation was based on circulating PMN ROS production. A total of 9 cows are needed to detect a delta of 30 ± 20% (mean ± standard deviation) in the production of ROS between postpartum and mid-lactation dairy cows (significance level = 0.05 and power = 80%), as previously reported by Rinaldi et al. [15].

### 2.3. Experiment 1: Effect of the Lactational Stage on Circulating PMN Production of NETs and ROS

In three replicates, three Holstein cows at distinct lactational stages were used for the present research aim. The experimental groups of triplets of cows consisted of virgin nulliparous heifers (<14 months old), late pregnancy cows (<1 month before the expected date of calving), postpartum cows (within 21 d after calving), and mid-lactation multiparous cows (between 90 to 120 d in milk with an average milk yield of 35 ± 2 kg per day). All of the included animals were metabolically and clinically healthy (having normal serum NEFA and BHB concentrations, unassisted calving, no fever, or other clinical diseases before or during the study period). The measurement of serum NEFA and BHB was performed using the Roche Cobas 6000 Clinical Chemistry Analyzer (Basel, Switzerland) with a c501 model. For NEFA 0.6 mM and 1.0 mM were used as thresholds for, respectively, the pre- and post-partum period. To diagnose hyperketonemia, serum BHB ≥ 1.2 mM during the postpartum was used as a threshold [4,16]. None of the included cows had peripheral levels above the NEFA or BHB thresholds before or after calving. All cows were housed in free-stall pens. Cows were fed a total mixed ration according to their physiological status and had ad libitum access to water. Lactating cows were milked twice per day in a separate milking parlor.

Cows were restrained in headlocks and blood samples (*n* = 5 per cow) were collected from their jugular vein using BD Vacutainer evacuated glass tubes containing EDTA (Franklin Lakes, NJ, USA). Samples were drawn at 7:00 a.m. (before feeding) and transported to the laboratory within 1 h. Then, PMN were isolated from the blood. Briefly, the blood samples were centrifuged at 4 °C at 600× *g* for 15 min. After centrifugation, plasma buffy coat and 1/3 of the upper layer of red blood cells were carefully discarded. The pellet was suspended in 8 mL ice-cold deionized water to lyse remaining erythrocytes, and the tube was gently flipped and mixed for 30 s. Osmolarity was adjusted by adding 0.05 M ice-cold phosphate-buffered saline (PBS, pH 7.3), and the tube was centrifuged at 200× *g* for 10 min. The supernatant was discarded, and the pellet was resuspended in 0.01 M PBS and washed twice (centrifuged at 500× *g* for 3 min). The final pellet was resuspended in 1 mL RPMI 1640 medium and stored in ice. The viability of PMN was evaluated via the trypan blue exclusion method [17]. To do so, 90 μL of the PMN suspension was transferred to a microcentrifuge tube, and 10 μL of a 4% trypan blue solution was added. Cell counting was conducted in triplicate using a hemocytometer chamber. Cell viability was calculated by the formula, cell viability (% viable cells) = number of living cells/(number of dead cells + number of living cells) × 100%. To determine morphology and purity, PMN were evaluated in smears using a Giemsa staining. Slides were analyzed with a 100 × objective and immersion oil by bright-field microscopy. Cell purity was calculated by the formula, cell purity (%) = number of PMNs/(number of white blood cells) × 100%. PMN were then diluted with RPMI 1640 medium into different concentrations according to the objective of each experiment (as described below).

#### 2.3.1. Morphological Observation and Quantification of NETs

PMN from each lactational stage group were diluted with RPMI 1640 medium, and 1 mL of the suspension (5 × 10^4^ cells/well) was added (in triplicate) into a 24-well plate in which poly-lysine cell climbing sheets were placed at the bottom in advance. Plates (covered with their lids) were cultured at 37 °C in 5% CO_2_ for 3 h to stimulate the cells to adhere to the poly-lysine cell climbing sheets. Next, sheets were fixed with 4% paraformaldehyde for 15 min and cleaned with 0.01 M PBS (pH 7.3) 3 times for 3 min each time. Then, 500 μL of 0.5% Triton X-100 (diluted with PBS) was added to permeate cells at room temperature for 20 min. Next, sheets were cleaned with PBS 3 times for 3 min each time, and in the last step, sheets were carefully dried up with absorbent paper. Then, sheets were stained with 500 μL of 5 μM Sytox Orange dissolved in PBS for 5 min at room temperature, and after that, sheets were washed with PBS 3 times for 3 min each time. Lastly, the sheets were taken out of the 24-well plates and placed on a glass slide. Samples were evaluated using a Carl Zeiss Fluorescence inverted microscope (Oberkochen, Germany). The average fluorescence intensity of 25 images at random fields per sample was analyzed by ImageJ software (V1.8.0.112) [18].

#### 2.3.2. dsDNA Quantification of NETs

The Quant-iT™ PicoGreen dsDNA Assay kit was used to quantify the level of NETs according to the manufacturer’s instructions. Briefly, PMN from each lactational stage group were diluted with RPMI 1640 medium, and 200 μL (5 × 10^5^ cells) were inoculated into 96-well plates (in triplicate) and cultured for 3 h at 37 °C and 5% CO_2_. At the end of the culture, a pipette was used to blow each well to break up the long dsDNA. Samples were then centrifuged at 500× *g* for 5 min, and 100 μL supernatant was transferred to a new black 96-well plate, and 100 μL Quant-iT™ PicoGreen working fluid to each well was added, gently mixed, and incubated in the dark at room temperature for 3 min. The fluorescence intensity of the samples was measured on a TECAN fluorescence microplate reader (Bern, Switzerland) at an excitation wavelength of 480 nm and an emission wavelength of 520 nm. The fluorescence intensity of each sample was calculated relative to the fluorescence intensity of blank samples (no cells).

#### 2.3.3. Detection of ROS

PMN from each lactational stage group were diluted with RPMI 1640 medium, and 200 μL (5 × 10^5^ cells) was subsequently inoculated into 96-well plates (in triplicate) and cultured for 3 h at 37 °C and 5% CO_2_. After incubation, the supernatant was discarded and 200 μL DCFH-DA solution diluted in 0.01 M PBS (pH 7.3) was added to each well, and the plates were incubated for 20 min at 37 °C and 5% CO_2_. Next, the cells were cleaned 3 times with PBS, and 200 μL PBS was finally added. Subsequently, the fluorescence intensity was measured on a TECAN Fluorescence microplate reader (Bern, Switzerland) at an excitation wavelength of 485 nm and an emission wavelength of 535 nm [19]. The fluorescence intensity of each sample was calculated relative to the fluorescence intensity of blank samples (no cells).

### 2.4. Experiment 2: Validation of a Model to Simulate the In Vitro Production of NETs and ROS by Circulating PMN

To validate a model which could simulate the in vitro production of ROS and NETs by circulating PMN, we performed titration curves using PMA and LPS as stimulators. The experimental setup included 3 virgin nulliparous heifers in 3 replicates. We decided to use nulliparous heifers due to their lower NETs and ROS basal production, as shown in Experiment 1 (see results section). Blood sampling, PMN isolation, viability, and dilution were performed as described earlier. LPS stimulation was conducted by adding 0, 1, 10, 25, 40, 50, 80 µg/mL of LPS to PMN suspensions (200 μL with 5 × 10^5^ cells). PMA stimulation was performed by adding 0, 10, 20, 40, 80, 100, 160 nM of PMA into PMN suspensions (200 μL with 5 × 10^5^ cells). The LPS and PMA concentrations used in our research model was based on the results obtained in previous studies [20,21]. All of the samples were run in triplicate. The quantification of PMN NETs (dsDNA quantification) and ROS were conducted after 3 h of incubation (5% CO_2_ at 37 °C) and measured using the same methods as described above.

### 2.5. Statistical Analysis

Statistical analysis was performed using the R language for statistical programming (R Core Team, Vienna, Austria, v3.6.0). The effect of the animal category (nulliparous, late pregnancy, early postpartum, or mid-lactation) on PMN ROS and NETs fluorescence intensities (AU) were fitted in mixed linear effects models (function lmer of the package lme4), including the replicate as a random effect. Moreover, we tested the effect of different supplementations of LPS (0, 1, 10, 25, 40, 50, or 80 µg/mL) and PMA (0, 10, 20, 40, 80, 100, or 160 nM) to nulliparous heifers’ PMN suspensions on ROS and NETs fluorescence intensities (AU), including the replicate as random factor. Model residuals were assessed using a scatterplot of the studentized residuals for homoscedasticity, a linear predictor for linearity, and a Shapiro-Wilk test for normality. The raw data were log2-transformed when the residuals of simple models were abnormally distributed (*p* < 0.05). For all of the transformed variables, the residuals were normally distributed (Shapiro-Wilk’s *p* > 0.05). Differences between levels of explanatory variables were assessed with the Tukey’s post hoc test. Results are expressed as least squares means and standard errors with their respective measured units. The level of significance was set at *p* ≤ 0.05.

## 3. Results

### 3.1. Experiment 1: Effect of the Lactational Stage on Circulating PMN Production of NETs and ROS

**Figure 1:**
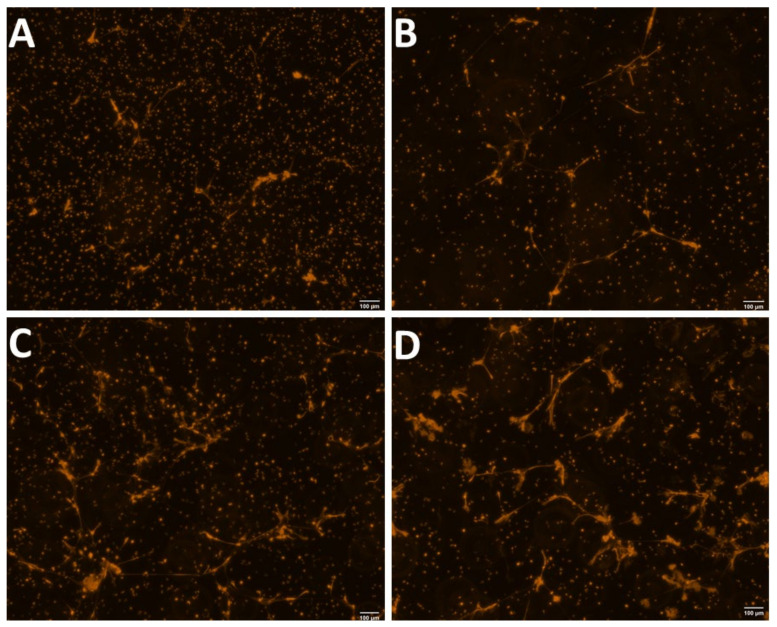
Representative images showing the quantification of the morphological observation of NETs evaluated via SYTOX Orange fluorescent staining in (**A**) nulliparous, (**B**) late pregnancy, (**C**) postpartum, and (**D**) mid-lactation Holstein cows.

The purity and viability of PMNs were >95% for all of the samples. Representative images of morphological observation and quantification of NETs production (evaluated via SYTOX Orange) from Holstein cows at different lactational stages are shown in Figure 1.

**Figure 2:**
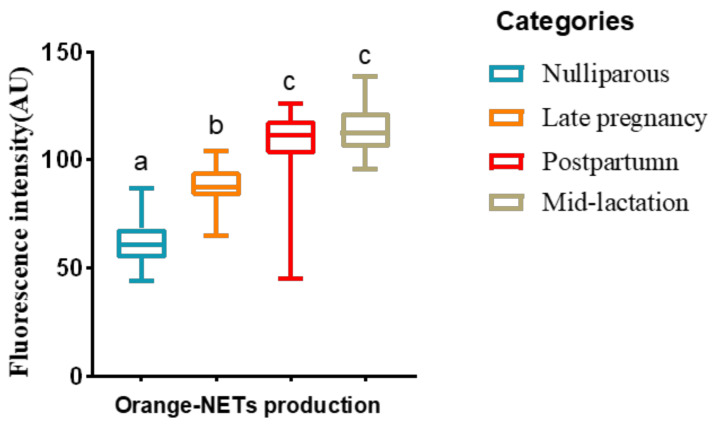
Boxplots showing the quantification of the morphological observation of NETs evaluated via SYTOX Orange fluorescent staining. Blood samples were collected from Holstein cows at different lactational stages. Different superscripts (a, b and c) represent *p* < 0.05.

The production of NETs in postpartum and mid-lactation cows were higher than that of nulliparous and late pregnancy cows (*p* < 0.05). However, the production of NETs in nulliparous cows was the lowest compared to all the other groups (*p* < 0.05; Figure 2).

**Figure 3:**
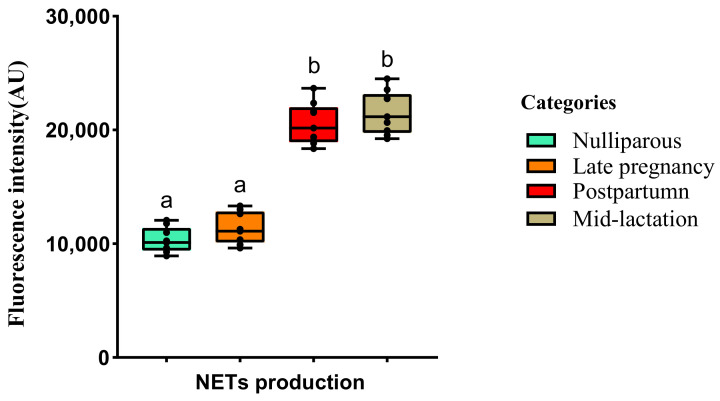
Boxplots showing the quantification of NETs evaluated via Quant-iT™ PicoGreen dsDNA assay kit. Blood samples were collected from Holstein cows at different lactational stages. Different superscripts (a and b) represent *p* < 0.05.

**Figure 4:**
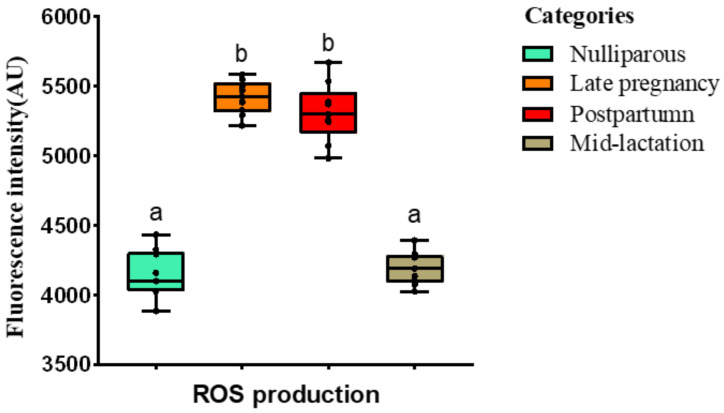
Boxplots showing the quantification of ROS evaluated via the fluorescent probe DCFH-DA. Blood samples were collected from Holstein cows at different lactational stages. Different superscripts (a and b) represent *p* < 0.05.

The quantification of NETs (via Quant-iT™ PicoGreen dsDNA) is shown in Figure 3. The quantification of NETs production was similar between nulliparous heifers and prepartum cows (*p* > 0.05). However, lactating cows (postpartum healthy and mid-lactation cows) produced more NETs than nulliparous heifers and prepartum cows (*p* < 0.05). The ROS values were lower in nulliparous heifers and mid-lactation cows than pre-and post-partum cows (*p* < 0.05), in which the the production of ROS was similar (*p* > 0.05; Figure 4).

### 3.2. Experiment 2: Validation of a Model to Simulate the In Vitro Production of NETs and ROS by Circulating PMN

**Figure 5:**
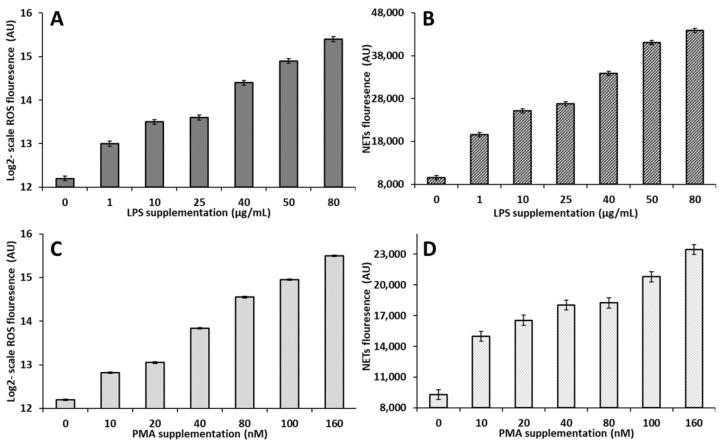
Validation of a model for the controlled in vitro production of PMN-derived ROS and NETs. PMN were isolated from the blood of nulliparous heifers and stimulation with LPS linearly increased (*p* < 0.05) the production of ROS and NETs (Figures (**A**,**B**)). Similarly, stimulation with PMA linearly increased (*p* < 0.05) the production of ROS and NETs (Figures (**C**,**D**)). ROS was evaluated via the fluorescent probe DCFH-DA and NETs were measured via the Quant-iT™ PicoGreen dsDNA assay kit. Values are shown as least square means with their respective standard errors.

For all of the samples, the purity and viability of PMNs were >95%. The stimulation of PMN isolated from nulliparous heifers with LPS linearly increased (*p* < 0.05) the product of both ROS and NETs (y = 448.27x + 5662, R^2^ = 0.9622 (Figure 5A) and y = 383.11x + 17280, R^2^ = 0.8653 (Figure 5B), respectively). Similarly, when PMN isolated from nulliparous heifers were stimulated with PMA, we observed a linear increase (*p* < 0.05) in the ROS (y = 264.23x + 4132.1, R^2^ = 0.997 (Figure 5C)) and NETs (y = 68.999x + 13302, R^2^ = 0.7875 (Figure 5D)) production.

## 4. Discussion

We found that circulating PMN isolated from dairy cows in the postpartum and mid-lactation stages produced more NETs in comparison to PMN isolated from late pregnancy cows and nulliparous heifers. The circulation of PMN of postpartum and mid-lactation cows may be primed to be in a pro-inflammatory state in comparison to those isolated from late pregnancy cows and nulliparous heifers. Previous studies used a physiological concentration of oleic acid or linoleic acid to stimulate the formation of NETs of PMN and extracellular ATP release in nulliparous heifers. It has been shown that 200 µM oleic acid or linoleic acid can induce a 2-fold formation of NETs in comparison to not-stressed PMN isolated from heifers [22]. In the present study, the NETs formation of PMN in transition cows was approximately 2-fold greater than that of heifers, while it is well known that the serum concentration of NEFA is significantly greater in transition cows than in heifers. Therefore, the present results may suggest that the concentration of NEFA may have primed the circulating PMN to produce NETs and therefore contribute to the typical immunomodulation that dairy cows experience in the transition period. As for mid-lactation cows, we do not have a clear hypothesis of which may be the trigger of the greater NETs formation; environmental, hormonal, social stress, or other managerial factors may have played a role. The increased production of ROS in peripartum dairy cows may explain the impairment of the immune system that transition cows experience, as it was previously described to affect phagocytosis and oxidative burst [23,24,25].

In the present study, the Sytox Orange and PicoGreen methods were used to quantitatively measure the amount of DNA that forms the backbone of the extracellular trap structures in vitro. Both methodologies are considered as two distinct but typical approaches to quantify the formation of NETs. A study in sheep recommends the use of two different dyes to quantify extracellular DNA and thus the formation of NETs, especially following a short-term incubation [26]. In the present study, we did not see too much difference between the two methods in terms of the quantification of DNA and hence NETs. However, images made by using the Sytox Orange dye led to the actual visualization of NETs, but the procedure is rather complicated, which tempts us to leave this method behind.

Earlier studies have shown differences in the vitality and functionality of PMN around parturition and mid-lactation. It is understood that there is not an important difference in the amount of PMN between healthy animals and sick cows before the onset of disease [27]. Therefore, the per-individual cell measurement of cell function is more representative for assessing the immunological capacity of PMN [24]. The impairments of the PMN function after parturition are often manifested as a severe cumulative deficit in the native defense mechanisms [28]. Meanwhile, phagocytosis and oxidative burst capacity are the PMN functions most consistently shown to be impaired in the week after calving [24]. In our study, the production of ROS by PMN isolated from postpartum cows was higher than that of heifers. The parallelism between the production of NETs and ROS suggests that the generation of NETs may be associated with the production of ROS, in the way that the production of ROS may be a mechanism or consequence of the formation NETs. However, in the present study, the production of ROS by the PMN of mid-lactation cows was somewhat low, although the formation of NETs in this group of cows was high. The latter suggests that other typical factors are more involved in the formation mechanism of NETs formation during mid-lactation. Therefore, more studies are warranted to explore the origin and dynamics in the formation of NETs by PMN of mid-lactation cows. In this context, it has also been reported that the transition from pregnancy to parturition in modern dairy cows is accompanied by a gradual decrease in PMN bactericidal capacity and respiratory burst activity in response to PMA stimulation, going from early to mid and late lactation [29,30]. Therefore, factors affecting the formation of NETs in postpartum and mid-lactation cows are multiple and probably closely related to their metabolism. It is, for example, well known that saturated fatty acids, which are usually high in the peripheral circulation of early postpartum cows due to the negative energy balance, activate toll-like receptor-4 (TLR4) signaling in numerous immune cells [31].

Healthy, nulliparous heifers are generally considered as experiencing a lesser degree of metabolic and environmental stress than transition or mid-lactation cows. The latter is confirmed by the first experiment of the present study in which it was shown that the the production of NETs by circulating PMN from nulliparous heifers is minimal in comparison to PMN isolated from animals from all of the other lactational stages. To validate the in vitro assessment of the inflammatory capacity of circulating PMN by measuring their NETs producing capacity, PMA and LPS were used as inflammatory stimuli to trigger NETs. PMA is the most frequently used inflammatory stimulus, with a 100% success rate for inducing NETosis [32]. LPS was used to stimulate the production of NETs because it can induce NETosis within 30 min. Interestingly, the increase in both NETs and ROS production was linear, with a strong correlation between the concentrations of LPS and PMA and the respective production of NETs. When performing in vitro experiments, LPS is frequently used to mimic infection and inflammation as in living organisms [33]. Similarly, PMA activates classical and conventional phosphokinase C and extracellular signal-regulated kinase signaling, mimicking NETs induction by bacteria and fungi [34]. An important finding in the present study is that the quantification of NETs is associated with the degree of infection and inflammation as a linear increase by LPS or PMA stimulation was found.

## 5. Conclusions

In the present study, we found that the in vitro formation of NETs and ROS by PMN isolated from dairy cows in early- and mid-lactation are higher than those isolated from late pregnant cows and nulliparous heifers. Typical factors associated with the physiological state of the animals may be able to prime the PMN to a pro-inflammatory state. The latter might contribute to the impaired immunological status of the animals in the early postpartum period. We furthermore validated an in vitro model to assess the formation of NETs and the production ROS by stimulating PMN by PMA (mimicking an inflammatory stimulus) and LPS (mimicking infection). We found a clear linear relationship between the concentration of the stimulants and the amounts of NETs and ROS production. Based on the observed data, we preliminarily conclude that when the level of infection (LPS) or inflammation (PMA) increases, there will be a linear, positive increase in NETs and ROS production. Therefore, we conclude that this model can be used to in vitro assess the effect of typical substances and influences on the formation of NETs and the production of ROS by PMN isolated from dairy cows.

## Data Availability

The data that support the findings of this study is available on the request from the corresponding author.
